# Stroke Evaluation in the Interventional Suite Using Dual-Layer Detector Cone-Beam CT: a First-in-human Prospective Cohort Study (the Next Generation X-ray Imaging System Trial)

**DOI:** 10.1007/s00062-024-01439-7

**Published:** 2024-07-25

**Authors:** Fredrik Ståhl, Håkan Almqvist, Åsa Aspelin, Jens Kolloch, Odett Ghalamkari, Vamsi Gontu, Dirk Schäfer, Peter van de Haar, Klaus-Jürgen Engel, Fred van Nijnatten, Åke Holmberg, Michael V. Mazya, Michael Söderman, Anna Falk Delgado

**Affiliations:** 1 Department of Neuroradiology, Karolinska University Hospital, Eugeniavägen 3, Stockholm, Sweden; 2 Department of Neurology, Karolinska University Hospital, Eugeniavaegen 3, 17176 Stockholm, Sweden; 3 Department of Clinical Neuroscience, Karolinska Institutet, Stockholm, Sweden; 4 Image Guided Therapy, Phillips Healthcare, Veenpluis 6, 5684 PC Best, The Netherlands; 5 Philips Research Hamburg, Roentgenstraße 24, 22335 Hamburg, Germany

**Keywords:** Cone-Beam CT, CBCT, CT, Dual-energy, Stroke, ASPECTS

## Abstract

**Purpose:**

Cone-beam CT in the interventional suite could be an alternative to CT to shorten door-to-thrombectomy time. However, image quality in cone-beam CT is limited by artifacts and poor differentiation between gray and white matter. This study compared non-contrast brain dual-layer cone-beam CT in the interventional suite to reference standard CT in stroke patients.

**Methods:**

A prospective single-center study enrolled consecutive participants with ischemic or hemorrhagic stroke. The hemorrhage detection accuracy, per-region ASPECTS accuracy and subjective image quality (Likert scales for gray-white matter differentiation, structure perception and artifacts) were assessed by three neuroradiologists blinded to clinical data on dual-layer cone-beam CT 75 keV monoenergetic images compared to CT. Objective image quality was assessed by region-of-interest metrics. Non-inferiority for hemorrhage detection and ASPECTS accuracy was determined by the exact binomial test with a one-sided lower performance boundary prospectively set to 80% (98.75% CI).

**Results:**

27 participants were included (74 years ± 9; 19 female) in the hyperacute or acute stroke phase. One reader missed a small bleeding, but all hemorrhages were detected in the majority analysis (100% accuracy, CI lower boundary 86%, *p* = 0.002). ASPECTS majority analysis showed 90% accuracy (CI lower boundary 85%, *p* < 0.001). Sensitivity was 66% (individual readers 67%, 69%, and 76%), specificity was 97% (97%, 96%, 89%). Subjective and objective image quality were inferior to CT.

**Conclusion:**

In a small single-center cohort, dual-layer cone-beam CT showed non-inferior hemorrhage detection and ASPECTS accuracy to CT. Despite inferior image quality, the technique may be useful for stroke evaluation in the interventional suite.

**Trial Registration Number:**

NCT04571099 (clinicaltrials.gov). Prospectively registered 2020-09-04.

**Supplementary Information:**

The online version of this article (10.1007/s00062-024-01439-7) contains supplementary material, which is available to authorized users.

## Introduction

Flat image detector cone-beam CT (CBCT) was first demonstrated in 2000 [1]. Today, the technique is widely used in interventional neuroradiology to assess anatomy and pathology [2, 3]. For stroke patients, CBCT in the interventional suite could be an alternative to non-contrast CT to shorten the door to thrombectomy time [4, 5]. However, CBCT is less sensitive than CT in identifying hemorrhage [3, 6] and early signs of ischemia [7–9], the extent of which typically is quantified by ASPECTS [10]. Yet, other studies indicate that latest generation CBCT may replace CT in the setting of acute stroke, with regards to ASPECTS score, hemorrhage detection accuracy and image quality indexes [11–13]. To date, one observational and one randomized single-center study have indicated that transferring patients with stroke symptoms suggestive of a large vessel occusion directly to the interventional suite may reduce intra-hospital time delays and improve functional outcome in those subjected to thrombectomy. A multicenter study is currently being conducted to verify the generalizability of the results [5, 14, 15].

Dual-energy CT virtual monoenergetic images (VMI) provide improved image quality compared to conventional CT [16–18], increasing the diagnostic accuracy in acute ischemic stroke [19–21]. In addition, radiation dose may be reduced [22]. Recently, a dual-layer detector dual-energy CBCT (DL-CBCT) prototype system was characterized, and a study on DL-CBCT VMI angiography reported non-inferior results to CT angiography under certain conditions [23, 24]. We hypothesized that non-contrast DL-CBCT VMI of the brain is sufficient for stroke diagnosis in the interventional suite. In this prospective non-inferiority study, we compare the hemorrhage detection accuracy and per-region ASPECTS accuracy of DL-CBCT to conventional CT in stroke patients. In addition, subjective and objective image quality metrics are reported in accordance with previous studies on dual-energy brain CT [16–18].

## Methods

### Study Design and Participants

The Next Generation X-ray Imaging System (NEXIS) trial was a single-center prospective cohort study conducted at a comprehensive stroke center (Clinicaltrials.gov identifier: NCT04571099). Patients with symptoms suggestive of an acute anterior circulation large vessel occlusion were triaged directly to the comprehensive stroke center as per clinical routine [25]. Participants aged 50 years or older with ischemic stroke of the anterior circulation or hemorrhagic stroke were consecutively enrolled during office hours from November 2020 to April 2021, after signing an informed consent. Initial imaging was performed according to clinical routine, typically including a non-contrast CT of the brain, multiphase CT angiography and CT perfusion (Canon Aquilion ONE, Canon Medical Systems, or Philips IQon, Philips Healthcare). Depending on eligibility criteria, participants were assigned to a specific inclusion group and imaged once or twice with DL-CBCT in an adjacent room (the same day as initial CT or/and one day after initial CT). Ischemic stroke patients were either included after intervention (inclusion group 3.II) or after a clinical decision had been made that the patient would not undergo thrombectomy (inclusion group 3.I). If the participant was imaged with DL-CBCT one day after initial CT, it was done in conjunction with a follow-up non-contrast CT. Eligibility criteria, inclusion groups and flow diagrams of study participation are presented in the supplementary information (see Eligibility Criteria and Supplemental Fig. 1). Participants also underwent a single phase DL-CBCT angiography, as reported in a previous publication [24]. The DL-CBCT system (Allura NEXIS Investigational Device, Philips Healthcare) was a commercial interventional C‑arm X-ray system (Allura Xper FD20/15, Philips Healthcare) fitted with a dual-layer 20 inch non-CE marked detector prototype [23]. CT and DL-CBCT scans had a comparable radiation dose (see scan details in Supplemental Table 1). Study protocol and informed consent forms were approved by the Swedish Ethical Review Authority (approval number: 2020-04467). Philips was the formal sponsor of the study with direct liability for the prototype DL-CBCT system, but was not involved in the data collection, interpretation or presentation of the results. The trial was supported by a grant from the European Commission (Horizon 2020, NEXIS-project, grant number 780026).

### Clinical Image Assessment

Pilot studies were carried out to determine the optimal VMI energy and noise reduction levels for the DL-CBCT images (see Pilot studies for optimal VMI energy selection in the supplementary information). Images of DL-CBCT 75 keV VMI and reference standard CT were randomized and evaluated independently by three neuroradiologists (Å.A., H.A., J.K.), each with over 9 years of experience. Images were presented one modality at a time in a single-sequence, two-period crossover design with a mean washout period of 4 weeks (range: 2–8 weeks before rating the next modality). Readers were blinded to modality and clinical information such as symptom lateralization (see Reader study details in the supplementary information). Hemorrhage and ASPECTS infarct assessment on CT constituted the reference standard, as judged by the individual reader. As the presence of hemorrhage or ischemic changes are typically evaluated by an independent radiologist, individual reader results were considered to best correspond with an actual clinical setting. A majority analysis is also presented, i.e. the result according to at least 2 out of 3 readers. The majority analysis corresponds to the most probable result within the given set of readers, mitigating potential inter-reader variability.

### Subjective Image Quality

Discrimination between gray and white matter was subjectively assessed in 17 supra- and infratentorial brain regions (ASPECTS regions and brain regions typically affected by artifacts) using 5‑point Likert scales (5: Excellent, fully diagnostic, 1: None, uncertain for diagnosis). The perception of intracranial structures and artifact presence were assessed in 26 brain areas, including CSF spaces and brain stem, in addition to the abovementioned regions (5: Excellent structure perception/No artifacts, 1: Structure not visible/Extensive artifacts, diagnostic evaluation impossible). See details about Likert scales and Regions evaluated in the supplementary information.

### Objective Image Quality

Objective image quality was assessed by circular ROIs on 5 mm thick axial slices in the asymptomatic hemisphere, in accordance with previous studies on dual-energy CT of the brain [16–18]. Noise, SNR and CNR for gray and white matter were determined using 25 mm^2^ ROIs in two cortical locations and their juxtacortical white matter at the level of the basal ganglia, in the thalamus and the posterior limb of the internal capsule. To assess the impact of artifacts, one 25 mm^2^ ROI was placed adjacent to the skull bone at the level of the basal ganglia (in the “subcalvarial space” [17]), and one 200 mm^2^ ROI in the interpetrous region of the posterior fossa. ROIs were automatically matched to identical positions in DL-CBCT and CT. See “Objective image quality assessment details” in the supplementary information for details on the calculations of noise, SNR, CNR and artifact indexes.

### Statistical Analysis

The sample size was calculated with regards to two prospectively defined performance goals with pre-defined thresholds for non-inferiority: the hemorrhage detection accuracy of DL-CBCT compared to CT (target accuracy 99.99%, lower boundary 80%) and diagnostic accuracy of DL-CBCT ASPECTS compared to CT ASPECTS (target accuracy 90%, lower boundary 80%). The ASPECTS endpoint was trait-based, encompassing each participant’s ten ASPECTS regions in the affected hemisphere. The target for hemorrhage detection was set not to miss any bleeding given a limited number of participants, whereas the lower boundary of the ASPECTS performance goal was set not to risk more than 20% false negatives or false positives (i.e. two ASPECTS areas). The minimum sample size was 20 participants for 95% and 90% power, respectively, whereas we estimated to enroll 29 participants to account for algorithm optimization and invalid data. The exact binomial test with one-sided 98.75% confidence intervals (adjusted for multiple endpoints) was used, with a significance level of *p* < 0.0125. See “Statistical considerations—sample size” in the supplementary information for details regarding the sample size calculation. Per-region ASPECTS agreement was assessed by the exact Fleiss Kappa [26]. The square-weighted Cohens Kappa was used to assess the numerical ASPECTS agreement between modalities.

The two-sided Wilcoxon signed-rank matched-pairs test and the two-sided paired t‑test were used for subjective and objective image quality comparisons. Bonferroni correction was applied to all image quality analyses with *p* < 0.05 considered significant. Statistical analyses were made in RStudio (v1.4.1103, The R Foundation for Statistical Computing). A biostatistician was consulted at all stages of the study.

## Results

**Fig. 1:**
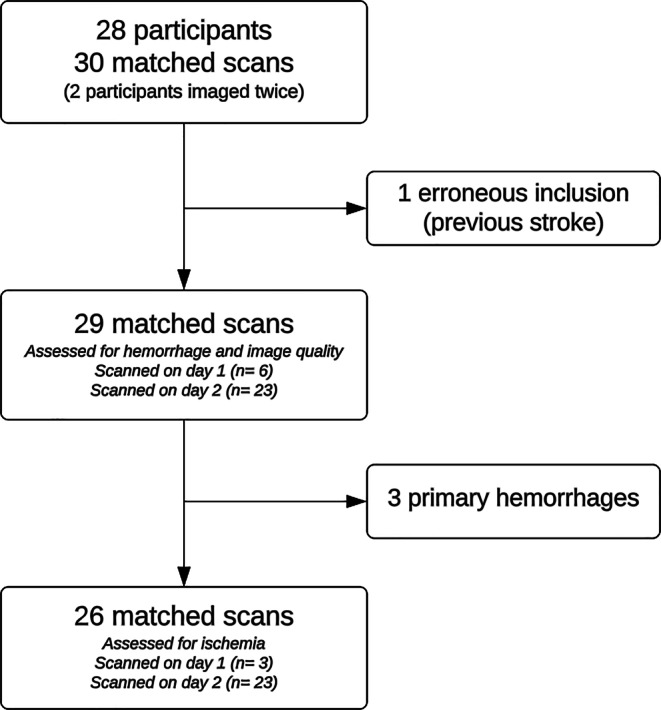
The flow diagram depicts which scans that were assessed for hemorrhage, image quality and ischemia, respectively

Of 28 consecutively enrolled participants, one had an old infarction and was excluded according to protocol. Two participants were imaged twice with DL-CBCT and CT (day one and two), and results from both matched scans were included in the dataset. Consequently, 29 complete matched DL-CBCT and CT image sets from 27 participants were included and analyzed for hemorrhage identification and image quality assessment (see flow diagram, Fig. 1). 26 matched image sets from 24 participants were evaluated for ischemic stroke, after excluding three participants presenting with hemorrhagic stroke.

The mean age was 74 years ± 9 and 19 participants were female (66%). The right hemisphere was affected in 17 participants (59%). Six matched scans (21%: 3 ischemic strokes, 3 hemorrhagic strokes) were performed on the day of symptom onset, the rest on day 2 (all ischemic stroke, post thrombectomy). Overall, the median time between CT and DL-CBCT was 17 min (IQR 12–28). Of the 26 scans used for ASPECTS region assessment, 24 (92%) presented with an occlusion of the intracranial internal carotid or proximal middle cerebral artery (M1 or M2 segment). Key demographic and clinical characteristics are presented in Table 1.

**Table 1:** Key demographic and clinical characteristics

Characteristic	All scans (*n* = 29)	Scans evaluated for ischemia (*n* = 26)
Patient age (y)^a^	74 ± 9	74 ± 9
Female	19 (66%)	19 (73%)
Right hemisphere affected	17 (59%)	17 (65%)
Scans performed on day 1	6 (21%)	3 (12%)
Scans performed on day 2	23 (79%)	23 (88%)
Time between CT and DL-CBCT (minutes)^b^	17 (12–28)	15 (12–22)
Hemorrhagic stroke	3 (10%)	0 (0%)
Intracranial internal carotid artery occluded^c^	3 (10%)	3 (12%)
M1 artery segment occluded^c^	12 (41%)	12 (46%)
M2 artery segment occluded^c^	9 (31%)	9 (35%)
M3 artery segment occluded^c^	1 (3%)	1 (4%)
M4 artery segment occluded^c^	1 (3%)	1 (4%)

Percentages of the total scans are presented in parentheses, except where indicated

*DL-CBCT* Dual-layer cone-beam CT

^a^Data are mean age in years ± SD

^b^Data are median (IQR)

^c^Vessel occlusion at admission, typically recanalized at the time of studied scans

### Hemorrhage Detection

Three primary hemorrhages and five hemorrhagic transformations of ischemic stroke were detected in the reference CT examinations (images of all hemorrhages are shown in Supplemental Fig. 2). Hence, eight matched scans with hemorrhages were included in the analysis (28% of all scans). Of these, two were small streaks of blood in the subarachnoid space. In the majority analysis, all eight hemorrhages were detected on DL-CBCT with 100% sensitivity and 100% specificity (100% accuracy, CI lower boundary 86%, *p* = 0.002). One reader missed a small bleeding in the subarachnoid space on DL-CBCT, resulting in 88% sensitivity and 100% specificity (97% accuracy, CI lower boundary 80%, *p* = 0.013) for the specific reader. There were no false positive hemorrhages.

### ASPECTS Assessment

**Fig. 2:**
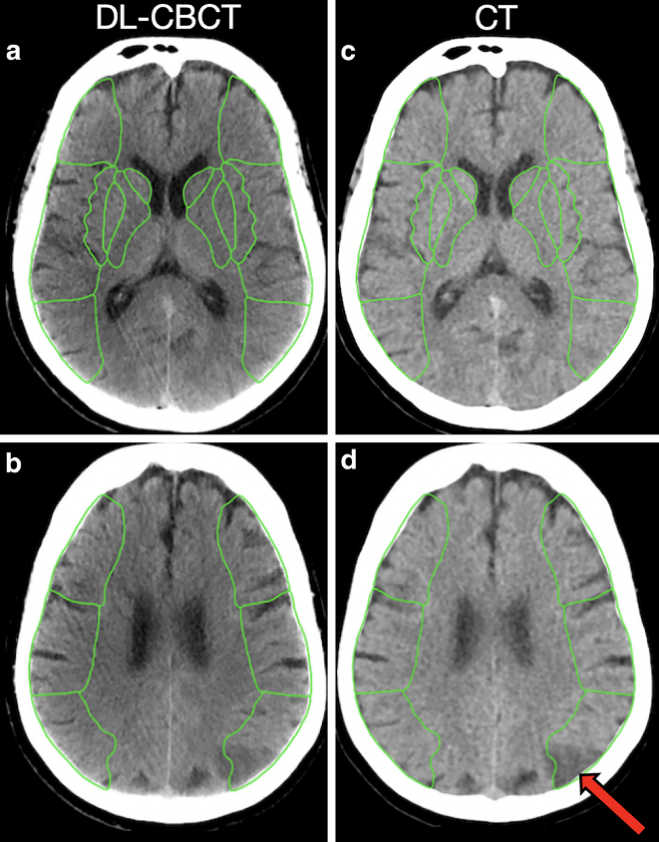
Axial non-contrast images of a participant with ischemic stroke. **a**–**d** shows ASPECTS regions boundaries at the level of the basal ganglia (**a** and **c**) and superior to the basal ganglia (**b** and **d**). **a**, **b** are DL-CBCT 75 keV images, **c**, **d** are CT images. There is an infarct in the left hemisphere M6 region (red arrow). All W/L: 75/25, 5 mm slices with average intensity projection. *DL-CBCT* Dual-layer cone-beam CT

In the per-region ASPECTS assessment (Fig. 2) all readers individually had a higher accuracy than the prospectively defined lower performance boundary of 80%, and the lowest accuracy was seen for Reader 3: 86%, CI lower boundary 80%, *p* = 0.010 (Table 2). The majority analysis showed 90% accuracy (CI lower boundary 85%, *p* < 0.001). Sensitivity was 66% in the majority analysis, however, individual readers performed slightly better (67, 69 and 76%, respectively). Specificity was 97% in the majority analysis (97%, 96% and 89% for each individual reader). Per-region Kappa was 0.49 for all readers (individually matched against each other 0.49, 0.42 and 0.54, all indicating moderate agreement, see Supplemental Table 2).

**Table 2:** Per-region ASPECTS assessment (*n* = 260)

Parameter	Majority	Reader 1	Reader 2	Reader 3
	TN: 200	TN: 189	TN: 210	TN: 179
	TP: 35	TP: 45	TP: 28	TP: 44
	FN: 18	FN: 20	FN: 14	FN: 14
	FP: 7	FP: 6	FP: 8	FP: 23
Accuracy	0.90 (0.85) *p* < 0.001	0.90 (0.85) *p* < 0.001	0.92 (0.87) *p* < 0.001	0.86 (0.80) *p* = 0.010
Sensitivity	0.66	0.69	0.67	0.76
Specificity	0.97	0.97	0.96	0.89

Data in parentheses are one-sided 98.75% confidence intervals

*TN* True negative, *TP* True positive, *FN* False negative, *FP* False positive

In the majority analysis, median CT ASPECTS was 9.5 (IQR 5–10) and DL-CBCT ASPECTS was 9.5 (IQR 7–10). All three ischemic strokes imaged on the day of symptom onset had ASPECTS 10 on the DL-CBCT and CT. The most commonly infarcted region was the insula, followed by the lentiform nucleus, M2 and M5, and the least infarcted regions were the M1, M3 and M4 (See Supplemental Table 3). The weighted Cohens Kappa between the numerical CT and DL-CBCT ASPECTS was 0.59 (0.42 unweighted, indicating moderate agreement, see Supplemental Table 4).

### Subjective Image Quality

The Likert scores from all readers were averaged (matched to each structure and scan evaluated). Median Likert scores for gray-white matter differentiation, structure perception and artifacts for all regions evaluated were 3.0 in DL-CBCT, compared to 4.0 or 4.3 for CT (Table 3). Results were inferior to CT, *p* < 0.001. DL-CBCT Likert scores were slightly higher when only assessing ASPECTS regions (median 3.0 or 3.3), but results were still statistically inferior to CT (Likert scores of ASPECTS regions in Supplemental Table 5). Frequency plots detailing the spread of all Likert scores are included in Supplemental Fig. 3. In addition, all individual regions were analyzed separately. DL-CBCT showed significantly lower scores for all individual regions compared to CT, except for structure perception of the lateral ventricles (boxplots detailing results of all individual regions in Supplemental Fig. 4).

**Table 3:** Average image quality scores for 3 readers (best score or least artifacts = 5)

Image quality parameter	DL-CBCT 75 keV VMI	CT
Gray vs white matter differentiation (*n* = 493)	3.0 (2.3–3.3) *p* < 0.001	4.0 (3.7–4.0)
Structure perception (*n* = 754)	3.0 (2.3–3.3) *p* < 0.001	4.0 (3.7–4.3)
Artifacts (*n* = 754)	3.0 (2.3–3.7) *p* < 0.001	4.3 (3.7–4.7)

Data presented are median (IQR). *P* values for DL-CBCT vs CT after Bonferroni correction

*DL-CBCT* Dual-layer cone-beam CT, *VMI* Virtual monoenergetic images

### Objective Image Quality

**Fig. 3:**
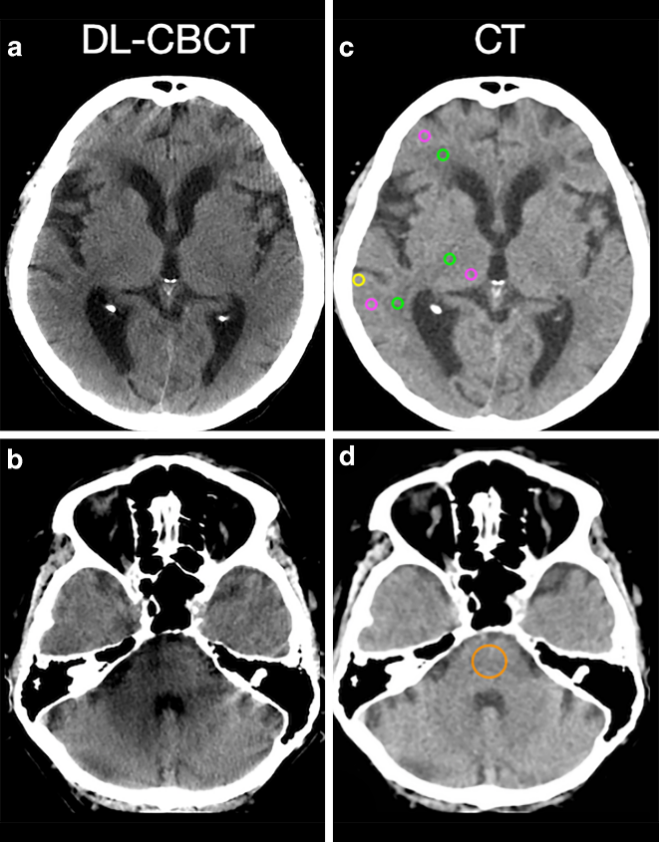
Axial non-contrast images of a participant with ischemic stroke. **c**, **d** show CT images with ROIs in the asymptomatic hemisphere and in the posterior fossa: gray matter (*Magenta*), adjacent white matter (*Green*), adjacent to the calvarium (“Subcalvarial”, *Yellow*) and in the posterior fossa (*Orange*). ROIs were automatically matched to the identical position in the DL-CBCT 75 keV images (**a**, **b**). All W/L: 75/25, 5 mm slices with average intensity projection. *DL-CBCT* Dual-layer cone-beam CT, *ROI* Region of interest

An example of typical ROI positioning is shown in Fig. 3. DL-CBCT showed a higher noise in all regions evaluated (*p* < 0.001), lower SNR in gray and white matter (*p* < 0.001), and a lower CNR between gray and white matter (*p* = 0.006) compared to CT (Table 4). The artifact indexes adjacent to the supratentorial skull bone (“subcalvarial”) and in the posterior fossa were higher in DL-CBCT (*p* < 0.001 and *p* = 0.004), indicating a higher degree of beam hardening artifacts compared to CT.

**Table 4:** Objective image quality indexes

Image quality index	DL-CBCT 75 keV VMI	CT
Gray matter noise	3.87 (1.05) *p* < 0.001	2.72 (0.42)
White matter noise	3.66 (0.89) *p* < 0.001	2.60 (0.39)
Gray matter SNR	6.76 (1.87) *p* < 0.001	13.23 (2.10)
White matter SNR	5.49 (1.57) *p* < 0.001	11.17 (1.87)
Gray vs white matter CNR	1.84 (1.06) *p* = 0.006	2.58 (0.92)
Subcalvarial artifact index	6.51 (1.82) *p* < 0.001	2.87 (0.81)
Posterior fossa artifact index	6.28 (3.02) *p* = 0.004	3.71 (0.72)

Data presented are mean (SD). *P* values for DL-CBCT vs CT after Bonferroni correction. “Subcalvarial” denotes a region adjacent to the skull bone at the level of the basal ganglia

*DL-CBCT* Dual-layer cone-beam CT, *VMI* Virtual monoenergetic Images, *SNR* Signal-to-noise ratio, *CNR* Contrast-to-noise ratio

## Discussion

This prospective single-center non-inferiority study compared dual-layer CBCT to CT in a setting of acute stroke. All hemorrhages were identified in the majority analysis (100% accuracy, one-sided CI lower boundary 86%, *p* = 0.002). However, one reader missed a small bleeding in the left Sylvian fissure (97% accuracy, CI lower boundary 80%, *p* = 0.013). The ASPECTS accuracy non-inferiority performance goal was met for all readers individually and in the majority analysis (90%, one-sided CI lower boundary 85%, *p* < 0.001). As expected, DL-CBCT showed significantly inferior image quality compared to CT in the subjective and objective analyses. The results suggest that DL-CBCT may be useful in the setting of stroke, despite an inferior image quality compared to CT.

Following recent advancements in conventional CBCT image reconstruction techniques, a retrospective study including 32 ischemic stroke patients reported only 0–25% sensitivity to identify acute ischemic changes [6]. Another research group conducted two monocentric studies comparing CBCT ASPECTS to CT ASPECTS: one retrospective study in 102 patients with varying pathologies showing 71% sensitivity and 94% specificity, and a prospective study on 24 stroke patients with 85% sensitivity and 83% specificity [11, 12]. One recent retrospective study on oblique trajectory CBCT (“sine spin”) in patients with varying pathologies stated 73% sensitivity and 95% specificity in reporting ischemic lesions in a binary (yes/no) approach [27]. The three latter publications did not present individual reader results and CBCT scans were typically done several hours before or after CT. Our study conducted solely on stroke patients with a median of 15 min between modalities shows comparable results to the three latter studies for both majority and individual reader results (66–76% sensitivity and 89–97% specificity). Our findings indicate that DL-CBCT has similar accuracy in assessing ischemic changes compared to previous studies on CBCT in our cohort with only stroke patients and a short time interval to the reference standard. Given that the prevalence of ischemic changes in our dataset is about 20%, it should be noted that the high rate of true negative results likely influences the accuracy. In this study, there was a higher sensitivity to detect basal ganglia infarcts compared to cortical infarcts (Supplemental table 3). Possibly, the basal ganglia are easier to appreciate compared to cortical areas due to the lack of beam hardening artifacts from the skull (which are typically more apparent in CBCT-imaging compared to CT-imaging). No false positives were noted for cortical areas. Our suspicion is that readers are more hesitant to label cortical areas as infarcted if there is uncertainty.

Per-region and numerical ASPECTS assessments are known to be highly variable between expert readers [28, 29]. Although we show high specificity (89–97%) for individual readers, sensitivity is lower (67–76%). This could explain why the interrater agreement is only moderate. In this study, there is similar agreement (i.e. moderate agreement) in the per-region analysis to the largest CT ASPECTS study to date (Kappa 0.49, 0.42 and 0.54 as compared to a mean per-region Kappa of 0.52 in the CT ASPECTS study [29]). Also, we show that the agreement of numerical ASPECTS in DL-CBCT compared to CT is similar to that between different readers in the CT ASPECTS study (Kappa unweighted 0.42 and weighted 0.59 compared to 0.39 and 0.56 in [29]). These findings reinforce that DL-CBCT can be used to assess ASPECTS with a similar variability to CT.

Three previous studies on CBCT evaluated the detection of intracranial hemorrhages. The first including 45 intracranial hemorrhages, showed 95–100% sensitivity and 100% specificity for intracerebral and intraventricular hemorrhages, but missed 50% of subarachnoid hemorrhages (*n* = 22) [6]. The second study reported 95–100% sensitivity and 97–100% specificity for all types of hemorrhages [11]. The third study showed 93% sensitivity and 90% specificity, with subarachnoid hemorrhage being the most challenging diagnosis [27]. These previous results are in line with our findings and indicate that although small subarachnoid hemorrhages remain a challenge for DL-CBCT, parenchymal hemorrhages (which account for the majority of hemorrhagic strokes) are typically detected. The small hemorrhage in the left Sylvian Fissure missed by one reader in our study occurred after treatment of an ischemic stroke.

Despite significantly inferior results in subjective and objective image quality indexes, it is interesting to note that the DL-CBCT gray and white matter SNR and CNR are similar to polyenergetic CT reconstructions at comparable dose levels 10 years ago [16]. For gray and white matter differentiation, the median DL-CBCT score of 3 indicates a “decent, diagnostic” image quality. The DL-CBCT median score of 3 for all intracranial structures indicates an acceptable structure perception and slight artifacts, with a slightly better result when only looking at ASPECTS regions. Our results indicate that DL-CBCT generally provides an acceptable intracranial image quality with slight artifacts, however with increased beam-hardening artifacts near the skull bone and in the posterior fossa compared to CT.

Limitations of this study include the monocentric study design and that the majority of our scans were acquired one day after stroke symptom onset. The accuracy to identify a “dense vessel sign” could not be studied as most participants had already undergone recanalization therapy. Moreover, posterior circulation stroke was not studied. Readers were blinded to clinical information such as symptom lateralization. However, as they were asked to assess the brain with regards to ASPECTS, they likely anticipated findings consistent with an anterior circulation stroke, adding potential confirmation bias. Since diffusion-weighted MRI is not part of our clinical routine, CT was used as the reference standard. Although the progression of time typically facilitates the distinction of ischemic changes, diffuse intracranial iodine contrast staining (due to blood-brain barrier leakage) may render the evaluation of ischemic changes more challenging [30]. As CT and DL-CBCT scans were acquired concurrently (typically a day after recanalization), both modalities were equally affected by potential iodine contrast staining. A selection bias was present as the participants were recruited during office hours and needed to agree to study inclusion independently (informed consent through a family member or two physician consent was considered ethically inappropriate). This rendered a relatively small cohort with limited infarct volumes, which impacts the generalizability of the results. The right hemisphere was affected in 65% of scans evaluated for ischemia, indicating a probable selection bias of participants without aphasia. Moreover, symptom time of onset, admission ASPECTS and NIHSS were not part of the eligibility criteria which further limits possible conclusions drawn from our results. Scoring of ASPECTS is known to be variable among readers, and in this study we chose to score participants at the ganglionic and supraganglionic level in accordance with the original definition [28]. This approach can miss information about ischemic changes in the adjacent ganglionic and supraganglionic territories. Only eight intracranial hemorrhages were included, limiting the generalizability of the results. We did not study the optimum VMI for different clinical tasks; instead one energy level was chosen based on pilot studies of perceived image quality. The selection of 75 keV VMI for DL-CBCT is in line with previous studies regarding overall perceived intracranial image quality [16–18], and the identification of ischemic stroke [19–21]. Future studies on DL-CBCT should consider to include more hemorrhagic patients, as well as patients with more extensive ischemic burden. Moreover, future studies may consider virtual-non-contrast and iodine images to enhance the diagnostic confidence of DL-CBCT.

## Conclusion

In a small single-center cohort of stroke patients, DL-CBCT showed non-inferiority to CT for hemorrhage detection and ASPECTS accuracy, despite inferior image quality. A notable study limitation was that the majority of scans were acquired after the hyperacute stroke phase (> 6 h), after recanalization therapy. Moreover, visualization of small subarachnoid hemorrhages after treatment remains a challenge. Our findings indicate that DL-CBCT may be useful for stroke assessment in the interventional suite.

## Supplementary Information

Supplementary information, tables, and figures

## Data Availability

The data that support the findings of this study are available from the corresponding author upon reasonable request.

## Abbreviations

CBCT: Cone-beam CT
DL: Dual-layer
VMI: Virtual monoenergetic images
