# Supplementary material for: Stroke Evaluation in the Interventional Suite Using Dual-Layer Detector Cone-Beam CT: a First-in-human Prospective Cohort Study (the Next Generation X-ray Imaging System Trial)
Source: Clin Neuroradiol. 2024 Jul 25;34(4):929–37. doi: 10.1007/s00062-024-01439-7 (PMC11564390; doi:10.1007/s00062-024-01439-7)
Supplement: Supplementary file 1 — Supplementary information, tables, and figures [file 62_2024_1439_MOESM1_ESM.pdf]

Supplementary Information (SI)

Article: Stroke evaluation in the interventional suite using dual-layer detector cone-beam CT: a first-in-human prospective cohort study (The Next Generation X-ray Imaging System trial)

Journal: Clinical Neuroradiology

Eligibility criteria

Also see flow diagram (Supplemental Figure 1)

Inclusion criteria

1. The patient has signed and dated the Informed Consent Form (ICF)
2. Age ≥ 50 years old
3. Clinical and radiological signs consistent with acute stroke
    - I. Patient diagnosed with ischemic stroke of the anterior circulation and not eligible for thrombectomy.
    - II. Patient diagnosed with ischemic stroke of the anterior circulation and subjected to thrombectomy.
    - III. Patient diagnosed with hemorrhagic stroke.

Exclusion criteria

1. Pregnant or breastfeeding women.
2. Previous stroke or parenchymal damage/defects in anterior circulation territories (only applicable for subjects included by criterion 3.I or 3.II).
3. Subject participates in a potentially confounding drug or device trial during the course of the study.
4. Participation in the study exposes the subject to risk, as assessed at the discretion of the treating physician.
5. All subjects who meet an exclusion criteria according to national law.
6. Subject or subject family member is a known Philips employee.

Patients were assigned to one inclusion group (I-III, depending on inclusion criterion #3 above), as depicted in Supplemental Figure 1. Approximately 90 suspected stroke patients were assessed for eligibility during the inclusion period.

Additional information about inclusion groups 3.I – 3.III

3.I patients (imaged twice with DL-CBCT) were originally intended to be part of a subgroup to evaluate the diagnostic accuracy to localize the arterial occlusion. Due to the scarcity of included subjects (n=3), this subgroup analysis was not performed. The results from the two DL-CBCT scans were both included, as described in the Results section. One patient only underwent the first day scan and was subsequently transferred to another hospital.

3.II patients (n=22) were imaged with DL-CBCT on day 2 to reduce risks related to iodine contrast media administration following CTA, CT perfusion and thrombectomy on day 1.

3.III patients (n=3) were imaged with DL-CBCT on day 1 since they were likely to be transferred to another hospital the same day as diagnosis.

Pilot studies for optimal VMI energy selection

Based on previous publications on ischemic stroke detection using VMI [19-21], a range of 50 to 90 keV VMI images at three different noise reduction levels were randomized and evaluated side-by-side by expert readers blinded to noise and energy level (F.S., H.A., V.G., M.S., A.F.D.). Images were ranked in order of preference with regards to image quality for acute stroke assessment. 75 keV images with a moderate noise reduction was considered appropriate for acute stroke assessment.

Reader study details

The study software default displayed axial and coronal image stacks in a 1 x 2 layout with 5 mm slice thickness (contiguous slices with average intensity projection). Slice thickness, viewing plane, window level and window width could be modified at the readers own discretion. Readers were asked to decide whether there was a hemorrhage, and then to identify infarcts according to ASPECTS.

ASPECTS regions were defined on a 1 cm thick slab at the level of the basal ganglia and on a second slab above the level of basal ganglia, in accordance with the original definition [28]. A dedicated software outlined the anatomical boundaries of the ASPECTS regions in all participants, to ensure that all readers assessed the correct regions on DL-CBCT as well as on the reference standard CT images.

The reader study software interface is displayed in Supplemental Figure 5 at the end of the supplemental material.

Objective image quality assessment details

Noise in gray and white matter were calculated as the mean standard deviation of the corresponding ROIs. The same calculation was applied for the ROIs adjacent to the skull bone and posterior fossa, but due to the impact of beam hardening artifacts by the adjacent skull bone these numbers are referred to as artifact indexes [16-18]. SNR was calculated as the mean CT number divided by the mean standard deviation (noise) of the ROIs. CNR between gray and white matter was calculated according to,

$$\text{CNR}(GM, WM) = \frac{\overline{GM} - \overline{WM}}{\sqrt{((\text{SD}(GM)^2 + \text{SD}(WM)^2)/2)}}$$

where $\overline{GM}$ and $\overline{WM}$ indicate the mean CT numbers and SD(GM) and SD(WM) are the standard deviations.

Likert scales

Gray and white matter differentiation

1. None, uncertain for diagnosis
2. Slight, limited diagnostic
3. Decent, diagnostic
4. Good, diagnostic
5. Excellent, fully diagnostic

Perception of intracranial structures

1. Structure not visible
2. Poor structure perception, blurring
3. Fair structure perception, suboptimal for confident diagnosis
4. Good structure perception, likely adequate for confident diagnosis
5. Excellent structure perception

Artifacts

1. Extensive artifacts, diagnostic evaluation impossible
2. Moderate artifacts, diagnostic evaluation impaired
3. Slight artifacts, may impair diagnostic evaluation
4. Faint artifacts, likely does not impair diagnostic evaluation
5. No artifacts

Regions evaluated

For all Likert evaluations:
M1, M2, M3, M4, M5, M6, Insula, Caudate Nucleus, Lentiform Nucleus, Internal Capsule
Frontobasal, Temporopolar, Cortex High, Occipital, Posterior Temporal
Cerebellum High, Cerebellum Low

Only evaluated with regards to structure perception and artifacts (in addition to above)
Ambient Cistern, Lateral Ventricle, 3rd Ventricle, 4th Ventricle, Foramen Magnum
Subcalvarial space
Mesencephalon, Pons, Medulla Oblongata

Statistical considerations – sample size

The sample size calculation for DL-CBCT ASPECTS was trait-based, encompassing the 10 ASPECTS regions of the affected hemisphere in each participant. To evaluate the diagnostic accuracy of non-contrast DL-CBCT compared to reference standard CT, it was estimated that a sample size of 137 was required to render a power of 90% (target accuracy of 0.90, performance goal lower boundary of 0.80, one-sided alpha of 0.025). The significance level of 0.025 was set with regards to using a one-tailed test for our main endpoint. The lower boundary of the ASPECTS performance goal was set not to risk more than 20% false negatives or false positives (i.e. two ASPECTS areas). For this endpoint, a minimum sample size of 14 participants (140 regions) was required.

The sample size calculation for DL-CBCT hemorrhage detection accuracy compared to reference standard CT was participant-based. It was estimated that a sample size of 20 was required to render a power greater than 95% (target accuracy of 0.9999, performance goal lower boundary of 0.80, one-sided alpha of 0.0125). The significance level was adjusted to 0.0125 to account for two secondary endpoints (one secondary endpoint is used for a separate sub-study of DL-CBCT angiography). The hemorrhage detection target was set to not miss any bleeding given a limited number of participants. The estimated sample size included both participants with hemorrhage and negative controls, with a threshold of at least a 0.25 prevalence. For this endpoint, a minimum sample size of 20 participants was required.

In total, the minimum sample size required was estimated to 20 participants, of which two-thirds have ischemia and one-third have an intracranial hemorrhage. Our estimated necessary sample size was 29 subjects, taking method optimization and potential invalid data into account.

Supplemental table 1: Scan details

	Canon Aquilion ONE	Philips IQon	Prototype DL-CBCT
Tube kilovoltage (kV)	120	120	120
Avg. tube current (mAs/mA)	[a] (auto modulation)	[a] (auto modulation)	310
Dose adjustment parameter	SD index 4.5 (0.5 mm)	DoseRight 36	[a]
Rotation time (s)	0.75 (Full rotation)	0.33 (Full rotation)	20.0 (200° rotation)
Nominal beam width (mm)	40 x 0.500	32 x 0.625	194.700
Pitch factor	0.625	0.343	[a]
Display FOV coronal x sagittal x axial (mm^3)	210.0 x 210.0 x *Z*	210.0 x 210.0 x *Z*	251.8 x 251.8 x 194.7
Slice thickness (mm)	0.50	0.80	0.66
Image matrix size	512 x 512	512 x 512	384 x 384
Reconstruction kernel	FC26	Filter UA	[b]
Reconstruction algorithm	AIDR 3D eStandard	$iDose^4$ level 2	[b]
Average CTDIvol (16 cm phantom)	43.4 mGy	44.1 mGy	[a]
Air kerma (in a 18 cm water phantom) [c]	[a]	[a]	57.6 mGy

Note: DL-CBCT = Dual-layer cone-beam CT, FOV = Field of view, Z = Length in Z axis.
[a] Not applicable
[b] Details of the prototype algorithm is described in a previous study [23]
[c] Air kerma in a 18 cm diameter plastic water phantom at the center of the scan length, measured in accordance with AAPM Task Group Report 111

Supplemental table 2: Per-region ASPECTS Kappa between readers

Kappa	DL-CBCT R1 vs R2 *κ*	DL-CBCT R2 vs R3 *κ*	DL-CBCT R1 vs R3 *κ*	DL-CBCT All *κ*
All regions (n=290)	0.49	0.42	0.54	0.49

Note: ASPECTS = Alberta Stroke Program Early CT Score, DL-CBCT = Dual-layer cone-beam CT, VMI = Virtual monoenergetic images, R = Reader.

Supplemental table 3: Scoring per-region, majority results

Performance	TN	TP	FN	FP	Sensitivity	Specificity	Accuracy
Caudate Nucleus	20	4	1	1	0.80	0.95	0.92
Internal Capsule	18	3	2	3	0.60	0.86	0.81
Insula	15	8	2	1	0.80	0.94	0.88
Lentiform Nucleus	16	8	0	2	1.00	0.89	0.92
M1	24	1	1	0	0.50	1.00	0.96
M2	19	3	4	0	0.43	1.00	0.85
M3	24	0	2	0	0.00	1.00	0.92
M4	23	1	2	0	0.33	1.00	0.92
M5	19	4	3	0	0.57	1.00	0.88
M6	22	3	1	0	0.75	1.00	0.96
**Total**	200	35	18	7			

Note: TN = True negative, TP = True positive, FN = False negative, FP = False positive.

Supplemental table 4: ASPECTS Score Kappa between modalities

Modality and Kappa analysis **(n=29)**	Majority
CT vs DL-CBCT VMI unweighted	0.42
CT vs DL-CBCT VMI weighted “equal”	0.54
CT vs DL-CBCT VMI weighted “squared”	0.59

Note: ASPECTS = Alberta Stroke Program Early CT Score, DL-CBCT = Dual-layer cone-beam CT, VMI = Virtual monoenergetic images.

Supplemental table 5: Average image quality Likert scores for 3 readers, only ASPECTS regions (best score or least artifacts = 5)

Image quality parameter	DL-CBCT 75 keV VMI	CT
Gray vs white matter differentiation (n=290)	3.0 (2.7–3.3)<br>p<0.001	4.0 (3.7–4.3)
Structure perception (n=290)	3.0 (2.8–3.3)<br>p<0.001	4.0 (4.0–4.3)
Artifacts (n=290)	3.3 (3.0–3.7)<br>p<0.001	4.3 (4.3–4.7)

Note: Data presented are median (IQR). P values for DL-CBCT vs CT after Bonferroni correction. DL-CBCT = Dual-layer cone-beam CT, VMI = Virtual monoenergetic images.

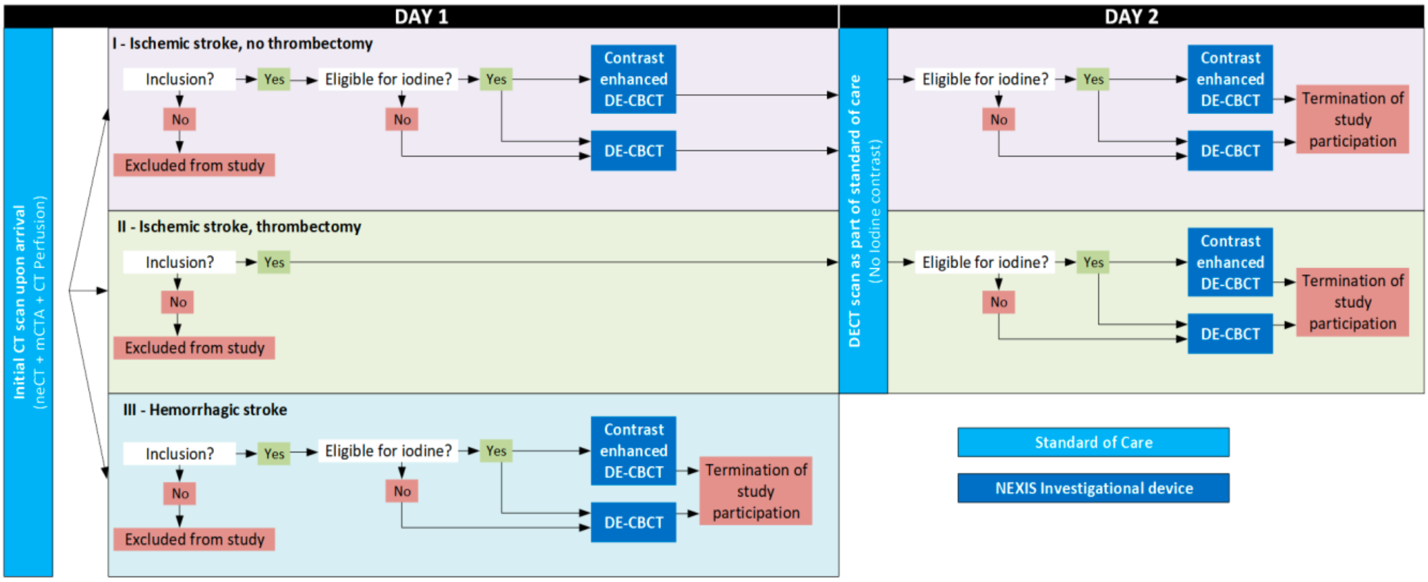

Supplemental Figure 1. Flow diagram: Participants were allocated to a specific inclusion group depending on eligibility criteria. DE-CBCT/DL-CBCT = Dual-layer cone-beam CT.

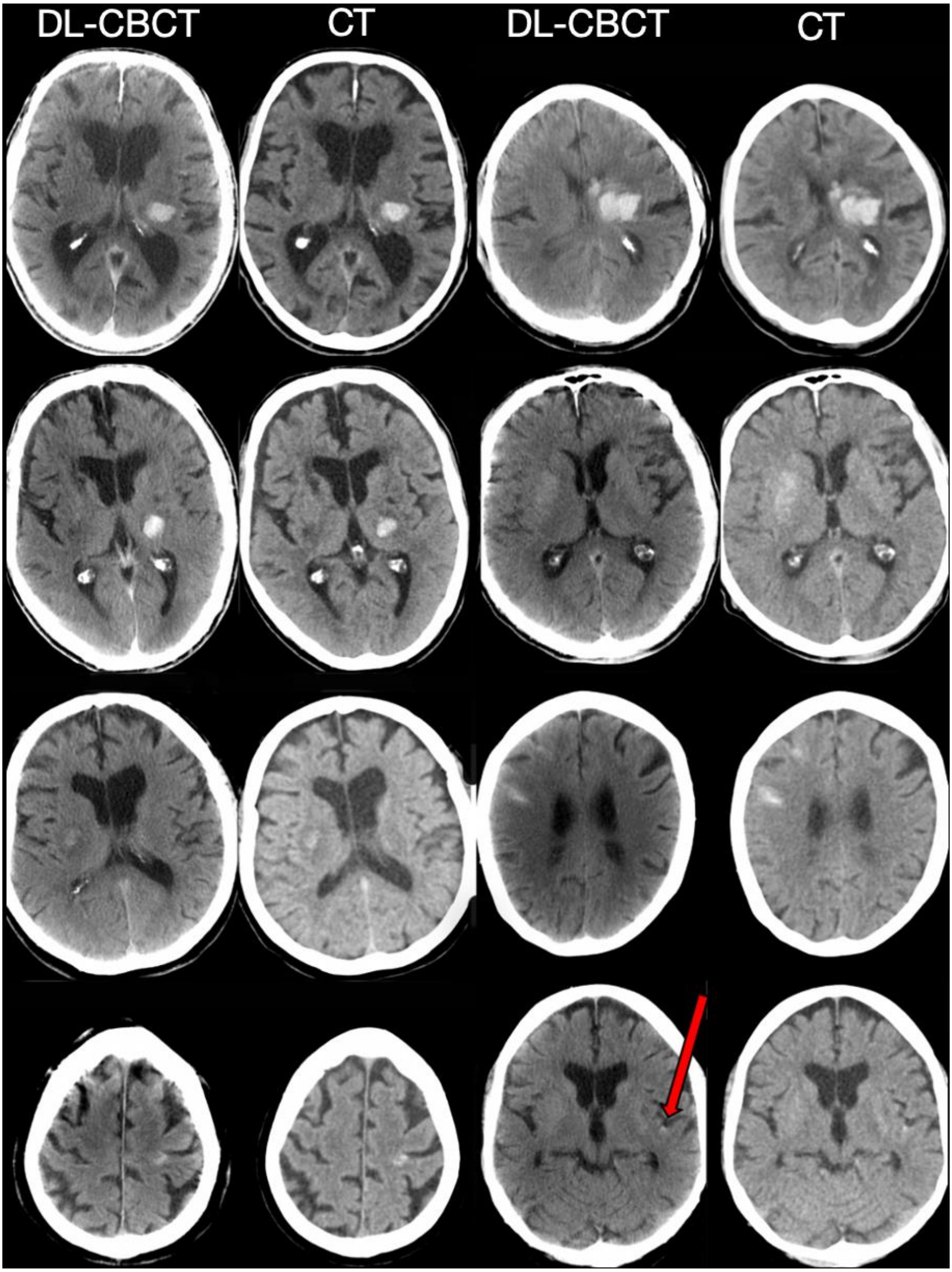

Supplemental Figure 2. All hemorrhages shown pairwise for comparison. Left: DL-CBCT 75 keV VMI, Right: CT. Red arrow shows a minimal bleeding that was missed by one reader (bottom right corner). DL-CBCT = Dual-layer cone-beam CT.

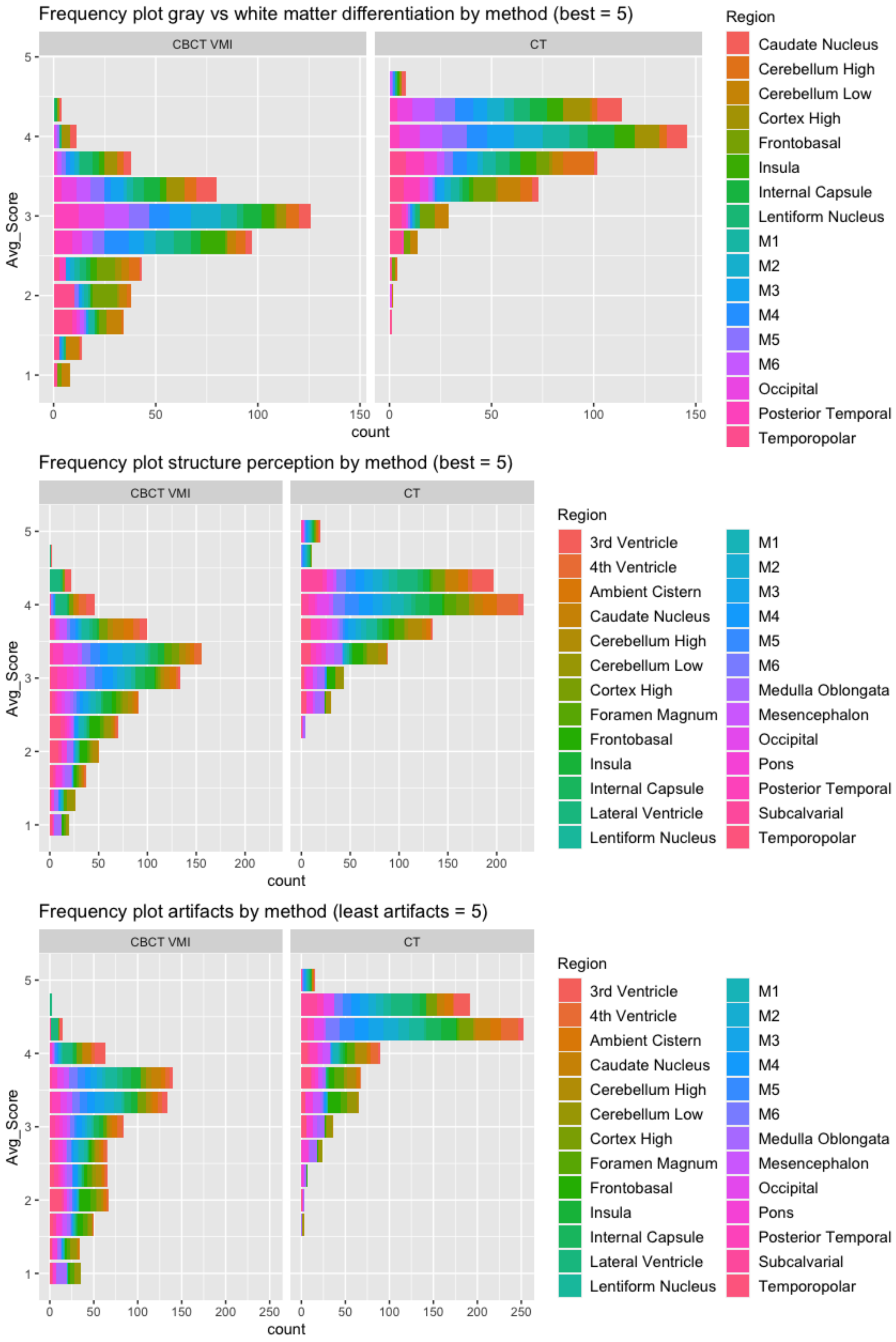

Supplemental Figure 3. Subjective image quality: Likert scores, frequency plots. CBCT VMI = Cone-beam CT virtual monoenergetic images.

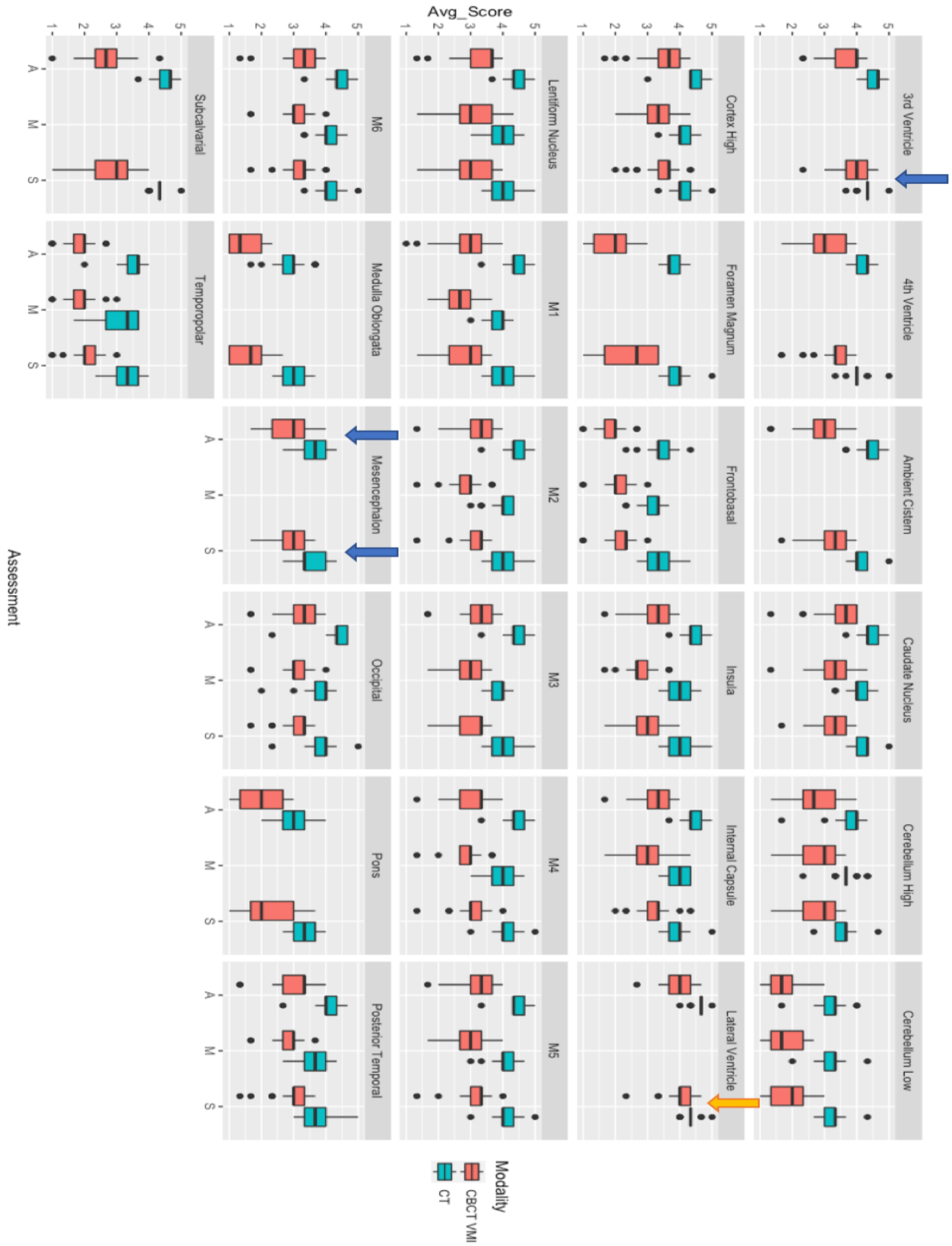

Supplemental Figure 4. Boxplots representing average scores for all regions evaluated. All results except those marked with arrows are significant of p<0.01 after Bonferroni correction. Blue arrows show significant results of p<0.05–0.01 after Bonferroni correction. Yellow arrow show non-significant result (only one: no significant difference in perception of lateral ventricles). A = Artifact presence; M = Gray-white matter differentiation; S = Structure perception. CBCT VMI = Cone-beam CT virtual monoenergetic images.

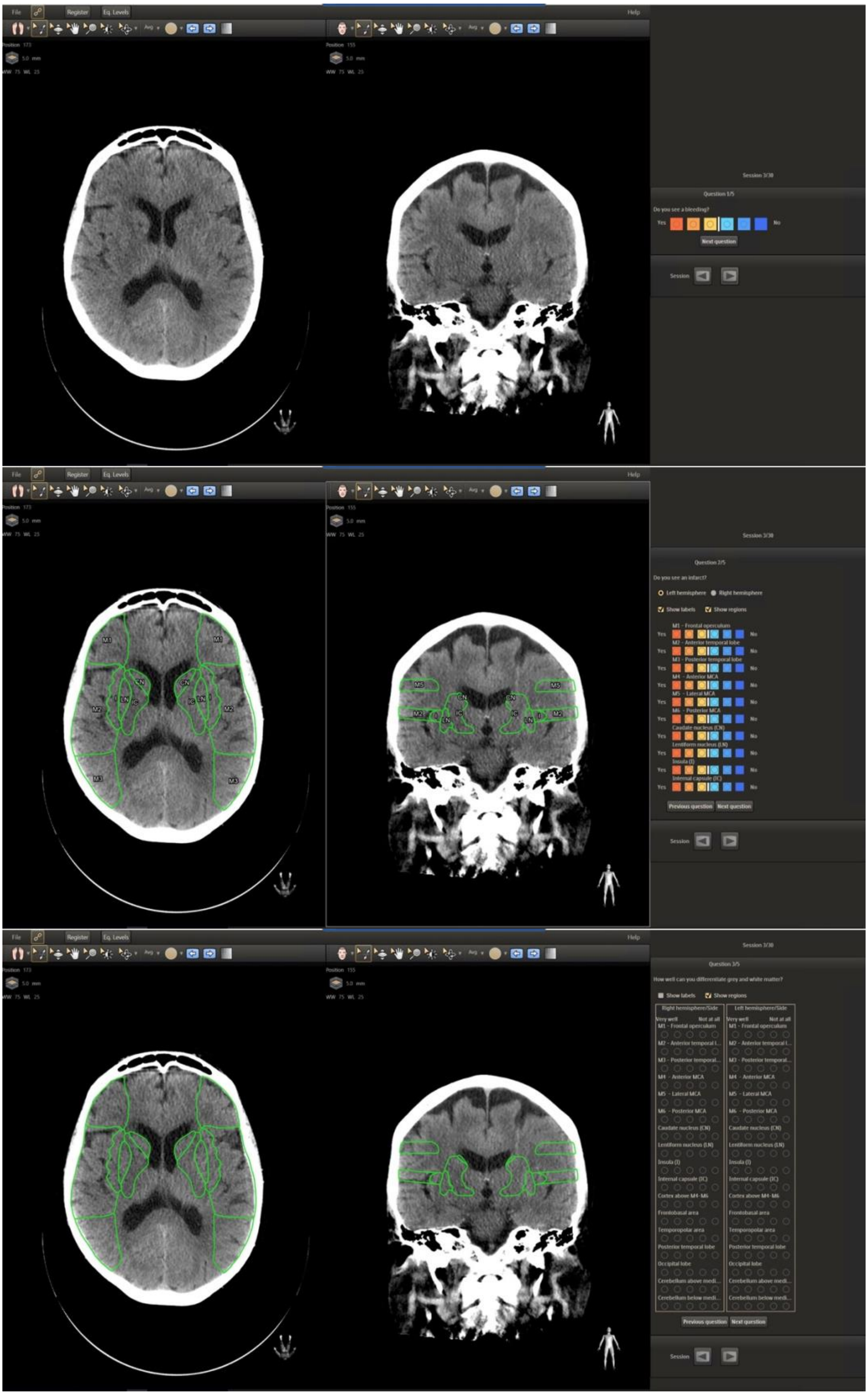
Session 3/30
Question 1/5
Do you see a bleeding?
Yes
No
Next question
Session
Session 3/30
Question 2/5
Do you see an infarct?
Left hemisphere
Right hemisphere
Show labels
Show regions
M1 - Frontal operculum
M2 - Anterior temporal lobe
M3 - Posterior temporal lobe
M4 - Anterior MCA
M5 - Lateral MCA
M6 - Posterior MCA
Caudate nucleus (CN)
Lentiform nucleus (LN)
Insula (I)
Internal capsule (IC)
Previous question
Next question
Session
Session 3/30
Question 3/5
How well can you differentiate grey and white matter?
Show labels
Show regions
Right hemisphere/Side
Left hemisphere/Side
Very well
Not at all
M1 - Frontal operculum
M2 - Anterior temporal l...
M3 - Posterior temporal...
M4 - Anterior MCA
M5 - Lateral MCA
Previous question
Next question
Session

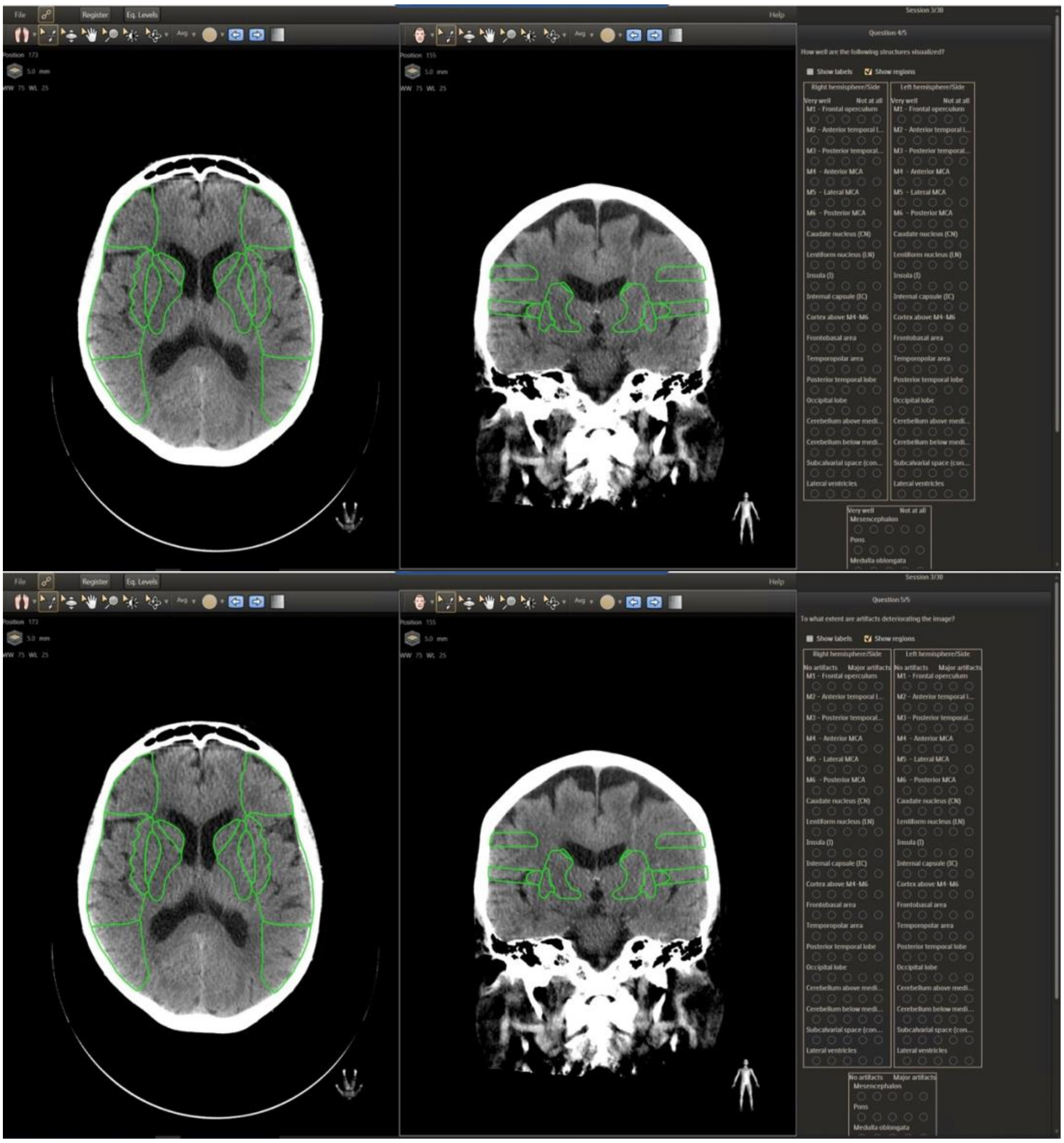

Supplemental Figure 5. Study software interface with DL-CBCT images. DL-CBCT = Dual-layer cone-beam CT.
